# The long and the short of priming in visual search

**DOI:** 10.3758/s13414-015-0860-2

**Published:** 2015-04-02

**Authors:** Wouter Kruijne, Martijn Meeter

**Affiliations:** Vrije Universiteit Amsterdam, Amsterdam, The Netherlands

**Keywords:** Visual search, Priming, Long-term memory, Implicit memory

## Abstract

Memory affects visual search, as is particularly evident from findings that when target features are repeated from one trial to the next, selection is faster. Two views have emerged on the nature of the memory representations and mechanisms that cause these intertrial priming effects: independent feature weighting versus episodic retrieval of previous trials. Previous research has attempted to disentangle these views focusing on short term effects. Here, we illustrate that the episodic retrieval models make the unique prediction of long-term priming: biasing one target type will result in priming of this target type for a much longer time, well after the bias has disappeared. We demonstrate that such long-term priming is indeed found for the visual feature of color, but only in conjunction search and not in singleton search. Two follow-up experiments showed that it was the kind of search (conjunction versus singleton) and not the difficulty, that determined whether long-term priming occurred. Long term priming persisted unaltered for at least 200 trials, and could not be explained as the result of explicit strategy. We propose that episodic memory may affect search more consistently than previously thought, and that the mechanisms for intertrial priming may be qualitatively different for singleton and conjunction search.

**Electronic supplementary material** The online version of this article (doi:10.3758/s13414-015-0860-2) contains supplementary material, which is available to authorized users.

## Introduction

A powerful factor determining where we look and what we attend is where we have looked and what we have attended before. The effects of our previous overt and covert shifts of attention on our current ones are often investigated by comparing visual search in which targets must be found with either the same features as on previous trials, or with different features. When compared to feature switch trials, feature repetitions have been found to shorten response times (RT) and decrease the amount of errors (Maljkovic and Nakayama, 1994). In addition, repetitions cause shorter saccade latencies (Becker, 2008; McPeek et al., 1999) and bias target selection (Brascamp et al., 2011a; Meeter and Van der Stigchel, 2013). Interestingly, such repetition effects have been found to affect vision largely out of the observers control (Maljkovic and Nakayama, 1994; Huang et al., 2004; Hillstrom, 2000). Collectively, these effects are called intertrial priming.

A wealth of priming research over the past decades (Kristjánsson and Campana, 2010) has as of yet failed to yield consensus on its underlying mechanisms. The primary dichotomy (Thomson and Milliken, 2013) appears to be between the *feature-weighting* account of intertrial priming and the *episodic retrieval* account. The feature-weighting account entails that the processing of a trial enhances the activation of those visual features that identify the target, and in addition may suppresses distractor features. This activation effectively changes how these features are ‘weighted’ on the next trial, which yields repetition benefits on subsequent trials that will decay over time. The feature-weighting view is intuitive, and the idea that trials can produce ‘lingering’ activity that affects subsequent performance is supported by several neurophysiological findings (Kristjánsson and Campana, 2010; Yeung et al., 2006; de Lange et al., 2013). Similarly, the idea that such weighting is subject to decay is in line with the observation that facilitation effects have been found to rapidly disappear over the course of some 5–8 trials (Maljkovic and Nakayama, 1994; Hillstrom, 2000), and that long intertrial intervals can attenuate or abolish priming effects (Maljkovic and Nakayama, 2000; Thomson and Milliken, 2012). Note that different properties of a search trial might independently contribute to priming effects: most notably, repetitions of the response, position, and target-defining feature on a search trial might independently produce repetition benefits or switch costs (Meeter and Olivers, 2006; Lamy et al., 2010; Tollner et al., 2008; Gokce et al., 2014). A mathematical implementation of the feature weighting account has been put forward by Maljkovic and Martini (2005), and updated in Martini (2010).

In contrast to the independent feature weighting view, the episodic retrieval account assumes that every trial is stored as a bound episodic memory, and that automatic retrieval of these memories affects performance on the current trial. Retrieval of matching trial traces facilitates the current trial, whereas nonmatching traces do not. Evidence supporting episodic retrieval over the feature weighting account came from the finding that repetitions of target features are not independent, but interact with intertrial repetitions or switches of response-features or task-irrelevant features, causing super- and under-additive priming effects respectively (Hillstrom, 2000; Huang et al., 2004). Evidence for this account is not limited to such interaction effects: recently, Thomson and Milliken (2013) reported that when trials were occasionally paired with a different task, these ‘rare’ trials would prime the next rare trial, 16 trials into the future. Not only does this ‘context dependence’ in priming contrast with independent feature weighting, but the prolonged time course of this effect also highlights the link with associative retrieval from memory.

Of note, these two views need not be mutually exclusive, and attempts to reconcile them through *hybrid accounts* have been put forward. For example, Lamy et al. (2011) found that the critical interaction effects that gave rise to the episodic retrieval view were only found when the task was difficult. To explain this finding, they emhpasized the dissociation between perceptual- and response priming, and concluded that feature weighting always affected the early perceptual stage – but that only when the task was difficult, observers would employ a strategy involving retrieval, which affects the response stage. Ásgeirsson and Kristjánsson (2011) similarly found that the interaction between the target feature and irrelevant features in the displays only appeared when search was difficult. They too proposed an account of priming that involves multiple stages (cf. Kristjánsson and Campana, 2010), and that episodic retrieval of past trials affected ‘late’ stages, yielding these interactions.

These effects are thus explained within both hybrid and episodic retrieval accounts by reference to retrieval of past trial episodes. However, debate has focused very little on how these results relate to the mechanisms of episodic retrieval. For example, an unexplored issue is whether episodic retrieval – which is generally probed at large time scales – can be reconciled with the observed time course of priming. To explore this issue, we have simulated priming experiments with a mathematical model of episodic memory (SAM, Search of Associative Memory, Raaijmakers and Shiffrin, 1981; Mensink and Raaijmakers, 1988; Raaijmakers, 2003), which implements some very general associative memory principles:

- memory for an item is acquired by forming traces in which the item is associated with the temporal context active during acquisition.
- The activation of such traces by the context active at retrieval determines the probability that an item is retrieved; and
- context randomly changes over time, gradually rendering items less accessible.

We subjected SAM to sequences of items that reflected search displays with alternating target colors. The details and results of these simulations are reported as Supplementary material (available online); to summarize, SAM could to a surprising extent capture results from multiple experiments exploring the time-course of priming (Maljkovic and Nakayama, 1994, Experiment 5; Brascamp et al., 2011b, Experiment 1). Its fits were comparable to those of a descriptive feature-weighting model with only short-term facilitation (Martini, 2010).

**Fig. 1:**
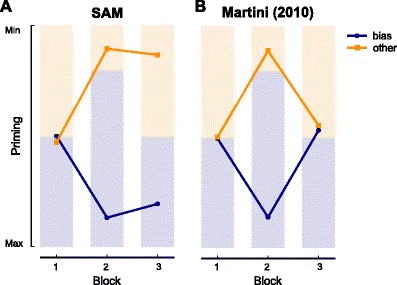
Prediction of priming effects by an episodic retrieval model (SAM, **A**) contrasted with the short-term feature weighting model of (Martini, 2010, **B**). Both panels show the results of one simulation of an experiment similar to the ones presented in this paper. Data points indicate the facilitation through priming predicted by both models (down indicates stronger priming) for either of the two colors in each block. Shading indicates the distribution of target colors in each block: in the bias block (2), one target color predominates, but in the neutral blocks both appear equally often. In both models, intertrial repetitions result in priming, but where the descriptive model predicts no differences between colors in all Neutral blocks, SAM predicts that bias blocks lead to durably faster responses for targets with the biased color

Further simulations with SAM revealed a prediction unique to the episodic retrieval account: as learning occurs on each presentation, more associated traces are formed for frequent than for infrequent items. In visual search, this implies that if one kind of target occurred more often than another, this results in easier retrieval of the associated trials, which results in stronger priming. This difference in priming will then persist long after the trial imbalance has disappeared (Fig. 1A). This prediction of *long-term priming* found with SAM can be generalized to any episodic retrieval account, as it results from instance-based learning, a mechanism shared by most models and theories on memory retrieval, which would probably make a similar prediction (for example Grossberg, 2013; Friston, 2005; Anderson, 2007, as well as applied to visual search: Navalpakkam and Itti, 2005; Logan, 2002; Shiffrin and Schneider, 1977).

In contrast, feature weighting accounts of priming as currently defined would not predict such long-term effects, as feature weighting is explicitly or implicitly assumed to be short-lived or subject to decay (Lee et al., 2009; Maljkovic and Nakayama, 2000; Chun and Nakayama, 2000; Kristjánsson and Campana, 2010). Although feature weighing accounts predict that a biased feature presentation results in cumulating priming effects Maljkovic and Martini (2005), simulations using the model of Martini (2010) showed that these effects would not persist after the bias has disappeared (Fig. 1B).

In this study, we present experiments to directly test these contrasting predictions: If feature biases yield robust facilitation that persists once the bias is long gone, this is in line with the predictions of memory retrieval, and thereby agree with an episodic retrieval account of priming. However if no long-term priming effects are found, this supports the idea that priming merely relies on a short lived facilitation mechanism. We first explore this question using the traditional priming of pop-out paradigm (Maljkovic and Nakayama, 1994). Additionally, since previous research has described that episodic retrieval only affect search when it is difficult and RTs are high, we also investigated the long-term priming prediction in a more difficult conjunction search task. We hypothesized that if retrieval is indeed heuristically recruited when search is difficult, possible long-term effects would be more pronounced in this task.

To preview the results: both tasks displayed comparable short-term priming effects. Long-term effects were absent in the simple singleton search task. In the conjunction search task, however, strong and persistent effects of the bias block propagated into the neutral blocks, producing long-term priming.

## Experiment 1A and 1B

### Method

#### Materials and stimuli

Stimuli were presented on a 21 in. LCD monitor at 120 Hz, in a dimly lit room at 70 cm viewing distance controlled by a chinrest. Each trial started with a central white (56.6 cd/m ^2^) fixation dot on a black (0.5 cd/m ^2^) background for 1200–1700 ms (randomly determined for each trial). Fixation was followed by a search display on the same background.

**Fig. 2:**
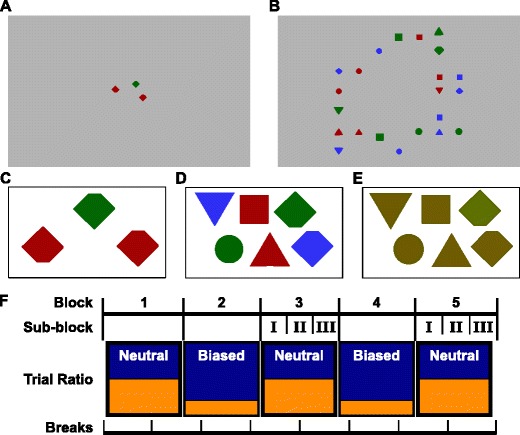
Stimuli and Design of the experiments. **A, B** An illustrative singleton search display from Experiment 1A and a conjunction search display from Experiment 1B, respectively. Note that in the experiments, the background was black rather than grey. **C,D,E** Exemplary illustrations of the stimuli used in Experiments 1A; 1&2B; 2A, respectively. In each, the target is the green(er) diamond in the top row. **F** General design, with alternating Neutral and Biased blocks. To explore the evolution of long-term effects, we divided neutral blocks of interest into sequential sub-blocks (**I**, **II** and **III**). Experiment 1A had two more blocks that continued this pattern. Participant breaks were distributed to not coincide with block boundaries

In Experiment 1A, the search display (Fig. 2A) contained three diamonds (sized 2.4°×2.4°) at locations randomly chosen from 12 equidistant positions at 4.05° eccentricity, none adjacent to another. Search displays contained two red (12.8 cd/m ^2^) and one green (13.3 cd/m ^2^) diamond or vice versa. All diamonds were missing a corner at the top or at the bottom (cut off at one-eighth of their height). Participants indicated which corner of the singleton colored diamond was missing.

In Experiment 1B (Fig. 2B), stimuli were distributed randomly over a 7×7 grid spanning 17.1°×17.1° excluding the center 3×3 positions (4.8°×4.8°). Stimuli were diamonds (as in 1A) or distractor shapes: triangles pointing up or down, rectangles, or circles, all with similar surface areas. Stimuli were colored red, green (as in 1A), or blue (13.1 cd/m ^2^). The target was a red or green diamond, and the 21 distractors were: twice all combinations of the three colors and distractor shapes, two blue diamonds, and one distractor item with a randomly chosen shape, colored red or green opposite to the target color. Features in all dimensions were thus approximately balanced in each display. Participants were instructed to search for the red or green diamond and respond to the missing corner.

In both experiments, participants responded by pressing ‘U’ or ‘D’ on a keyboard to indicate whether the missing corner was at the top (Up) or bottom (Down) of the target diamond. After the response the display was cleared and the next trial started. Error responses were followed by a 90ms tone.

#### Design and procedure

Both experiments started with 10 practice trials with random target colors, followed by alternating ‘Neutral’ and ‘Bias’ blocks, 200 trials each (see Fig. 2F). In Neutral blocks, targets were randomly red or green on 50 % of the trials. Repetition- and switch-trials were balanced per block, one occurring at most 5 % more often than the other. In Bias blocks, 80 % of the trials had the bias color as the target (red or green, counterbalanced across participants). Experiment 1A had seven blocks, Experiment 1B had five to compensate for longer search durations. Both experiments took about one hour.

Participants were instructed to respond as fast as possible while maintaining high accuracy (≈ 90 *%*). Every 125 trials, participants had a break and received feedback regarding their accuracy and average RT. These breaks did not overlap with the transitions from bias to neutral blocks, to prevent that these transitions were conspicuous.

To explore potential long-term priming effects on a finer timescale than whole blocks of 200 trials, we also split each Neutral block following a bias block into three sequential sub-blocks (**I**,**II** and **III**), each 66 or 67 trials. This allowed us to explore whether, for example, a long term priming effect was present, but only immediately after the bias, or whether it would not decay at all throughout the neutral blocks. Additionally, we were interested in participants’ subjective experience of the bias manipulation, and to what extent this could underly any observed long-term effects. We inquired about participants’ experience immediately after completing the experiment, and asked them to indicate on a line what they thought the distribution of red and green target trials had been. We rescaled these estimates to a [−1,1] domain, where positive numbers indicate estimates in the direction of the bias, and -1 and 1 reflect having observed only one target color.

#### Participants

Participants in all experiments were students from the Vrije Universiteit Amsterdam. All reported normal color vision, and otherwise normal or corrected-to-normal vision. They were naive with respect to the purpose of the experiment or the trial imbalance manipulation. Participants received course credits or monetary compensation. Informed consent was obtained prior to the experiment, in accordance with the guidelines of the Helsinki declaration.

Data were collected until the stopping criterion was met (discussed below). Participants were excluded from analysis beforehand if they were incorrect on over 15 % of all trials in the experiment (none in these two experiments), or if their average RT was more than 3SD away from the group mean (one in Experiment 1A). In Experiment 1A data from 31 students was included (26 female, ages 18–28, M = 21.4, SD = 3.1). Experiment 1B had 26 participants (16 female, ages 17–30, M = 20.7, SD = 3.3).

#### Trial inclusion and color-correction

For the analyses, only the data from neutral blocks were considered. We discarded trials immediately following breaks, error trials and outlier trials (RTs over 2.5SD away from the participant mean). These criteria discarded 7.7 % of 800 Neutral trials in Experiment 1A (on average per participant 4.3 % errors and 2.7 % outliers). In Experiment 1B this was 6.1 % of 600 Neutral trials (2.9 % errors and 2.7 % outliers).

Our goal was to investigate whether bias blocks facilitated search on subsequent trials with the bias color in subsequent neutral blocks. To obtain a measure isolating this effect, we attempted to correct for performance differences for both colors that participants might have *a priori*. Such differences would be reflected in the data from the first (Neutral) block. We attempted to correct for these color differences while taking into account the learning effect over blocks. First, we standardized RTs separately per block via a z-transform ($\textnormal {z}RT = \frac {RT-M}{SD}$). In the first block, we then computed the difference in zRT of bias- and other- color trials compared to the mean. We adjusted for this a priori difference in the z-scores of both trial types in all blocks. The resulting color-corrected zRTs are used in the analyses outlined below.

**Fig. 3:**
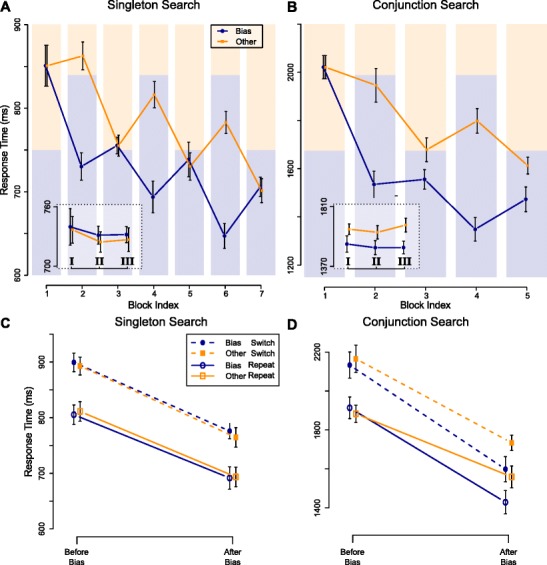
Color corrected RTs in Experiments 1A (left) and 1B (right). Shaded bars indicate relative target color proportions in each block. Error bars reflect 95% Cousineau-Morey confidence intervals (Baguley, 2011). The insets depict the evolution of the priming effect within neutral blocks, i.e. in sub-blocks **I**, **II** and **III**. **A** In singleton search, the color bias speeds responses to the bias color, but only during the bias blocks. **B** In conjunction search, the RT difference transfers to the neutral blocks, and does not decay within the neutral block itself. **C,D** In both tasks, repetition and switch trials reveal short term priming, both before and after the bias. In conjunction search an additional long-term priming effect is found after the bias

**Fig. 4:**
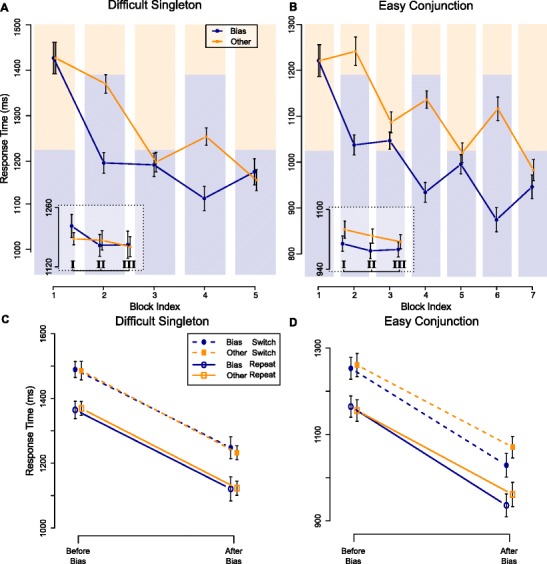
Results for Experiments 2A (left) and 2B (right), as in Fig. 3. Although the overall RTs in both experiments overlap, the bias color had no effect in subsequent neutral blocks in difficult singleton search (**A**) but was persistent in easy conjunction search (**B**). The insets reveal that again, long-term priming did not change or decay within neutral blocks. **C** Again, singleton search shows strong intertrial priming effects on both colors, unaffected by the bias block. **D** In conjunction search these effects are found both before and after the bias blocks, but after the bias, in addition a long-term priming effect is found

The zRTs do not convey information on overall RTs per task. In Figs. 3 and 4, we therefore plot values after they have been transformed back to RTs via an inverse z-transform, again using the mean and standard deviation per block. Similar figures of uncorrected RTs are provided as Supplementary material (available online).

#### Statistical analysis

To quantify the evidence for a long-term priming effect as well as evidence against it, we analyzed our data with Bayes Factors (*BF*). These quantify the relative evidence for statistical models; for example, *B* *F*_A,B_ = 3 implies the data are three times more likely under model $\mathcal {M}_{A}$ than under model $\mathcal {M}_{b}$ (which inversely implies $BF_{B,A} = \frac {1}{3}$). We used the ‘BayesFactor’ R-package to compute *BF*s for repeated measures analysis of variance-designs through Markov-Chain Monte Carlo (MCMC) sampling (Morey and Rouder, 2014; Rouder et al., 2009; Rouder and Morey, 2012; Rouder et al., 2012).

We collapsed data across post-bias neutral blocks, and computed the *BF* among four models, explaining zRT based on factors ‘repetition type’ (feature repetition- or switch trials), ‘target type’ (bias-colored trials and other-colored trials) and ‘sub-block’ (**I**, **II**, **III**):

- $\mathcal {M}_{1}:$ repetition type;
- $\mathcal {M}_{2}:$ $\mathcal {M}_{1}$ + target type;
- $\mathcal {M}_{3}:$ $\mathcal {M}_{1}$ + target type × sub-block; and
- $\mathcal {M}_{4}:$ $\mathcal {M}_{1}$ + target type + target type × sub-block.

All models included ‘participant’ as a random factor. The four models reflect different hypotheses on long-term priming effects: all models include the simplest model ($\mathcal {M}_{1}$) that assumes only (short-term) repetition effects of the preceding trial . Of primary interest was whether the data support an additional main effect of target type, indicating persistent long-term priming ($\mathcal {M}_{2}$). A third hypothesis explores whether the bias might have a long-lasting facilitating effect, but that this dissipates over the neutral block, modeled by an interaction effect of target type × sub-block ($\mathcal {M}_{3}$). Model $\mathcal {M}_{4}$ explored evidence for both the main effect (from $\mathcal {M}_{2}$) and the interaction from ($\mathcal {M}_{3}$). A definition of and motivation for the priors placed on all effects in these models can be found in (Rouder and Morey, 2012). All reported standardized effect sizes and their 95% confidence intervals (CI) are estimated from a posterior distribution based on 10^5^ MCMC samples.

For both experiments data were collected until either one of $\mathcal {M}_{2,3,4}$ was preferred over $\mathcal {M}_{1}$, or $\mathcal {M}_{1}$ over all models, with *B* *F*>10.0 – provided that the underlying model parameters were in line with the hypothesis they reflected. For example, if the best fit of $\mathcal {M}_{2}$ would attribute a long-term priming effect to the other color rather than the bias color, it would not be in line with the long-term priming hypothesis. In such cases one-sided Bayesian t-tests were used, collapsing the data over all conditions to contrast bias- with other-colored trials, to reject or accept the models, again at *B* *F*>10.0. In the results, only the *BF* between the two best-fitting models is given, unless otherwise indicated. Note than unlike significance testing using *p*-values, Bayesian inference does not depend on the assumption of a predefined sample size. As a result, collecting data until preset criteria on the relative evidence for statistical models are met is not only a valid way of hypothesis testing, but arguably a preferable one (Wagenmakers, 2007; Wagenmakers et al., 2012).

Due to the instructions and inclusion criteria, accuracy in this task was expected to be high, and no particular effects were expected due to ceiling performance. Accuracies were analyzed with the same models as RTs, but including a model that assumed no effects other than random variation across participants ($\mathcal {M}_{\textnormal {null}}$). Any further analyses will be introduced below as they are employed.

### Results

#### Experiment 1A

##### Long- and short-term priming following a bias

RTs for both target colors over the different blocks in Experiment 1A are depicted in Fig. 3A. The plot reveals an overall learning effect over blocks, as well as clear differences in performance for both colors in the bias-blocks. Critically, the plot reveals no clear differences between both colors in the neutral blocks, suggesting that the color bias had no long-lasting effect. The inset of Fig. 3A shows RTs for the different colors over sub-blocks. This plot shows that even early in the block (in sub-block **I**), no difference is found. This again argues against a long-lasting priming effect.

In line with these observations, model $\mathcal {M}_{1}$, including only short-term repetition effects, was best supported by the data, with *B* *F*_1,2_ = 12.0. This argues against long-lasting effects induced by the bias blocks. In line with past findings in similar tasks, we did find strong evidence for short-term repetition effects: excluding them from the best fitting model (yielding $\mathcal {M}_{1-\text {rep}}$, effectively $\mathcal {M}_{\textnormal {null}}$) resulted in a *B* *F*_1,1−rep_>1000. Repetition had strong benefits on RT, indicated by the distribution of the difference of repetition versus switch effects (M = 0.44, CI: [0.40,0.49]). Figure 3C illustrates these repetition effects, and suggests that they are similar before and after the bias manipulation.

##### Accuracy

As expected, overall accuracies were high (M = 95.7%, SD = 2.5%). The best model to predict accuracies was $\mathcal {M}_{1}$, suggesting performance on repetition trials was slightly better than on switch trials (effect size M =0.008, CI: [0.002,0.015]). However, with $BF_{1, \mathrm {M_{null}}} = 2.3$, evidence for this repetition effect was not convincing.

##### Target color distribution estimates

Although no bias effects were found, participants subjective experience was in general in line with their feature bias, judging by their distribution estimates (M = 0.09, SD = 0.15). One sided Bayesian t-tests revealed strong evidence that scores were overall higher than 0 (*B* *F*_+,0_ = 34.9). We explored whether participants’ experience of the bias could serve as a predictor for their long-term priming effect (the difference between bias color zRTs and other color zRTs), but a Bayesian regression analysis (testing *β*≠0) showed evidence for the absence of a correlation, both when effects are collapsed over all neutral blocks (*B* *F*_0,*β*_ = 5.2) as when only the last block was considered (*B* *F*_0,*β*_ = 5.2).

#### Experiment 1B

##### Long- and short-term priming following a bias

The pattern of RT results of Experiment 1B is shown in Fig. 3B. Again, performance increased over blocks, and differences between colors during the bias blocks are clear. Critically, this color difference now appears to persist in subsequent neutral blocks, suggesting a robust long-term priming effect. Moreover, the difference between colors is consistent across sub-blocks, as depicted in the inset, which indicated that the long-term priming effect did not dissipate over the course of a neutral block.

The analysis for Experiment 1B was in line with these observations, as model $\mathcal {M}_{2}$, which includes main effects of repetition- and target type, vastly outperformed the other models. The *B* *F*_2,1_>1000 suggested very strong evidence for a main effect of target type in this task. $\mathcal {M}_{2}$ was also preferred over $\mathcal {M}_{4}$ with *B* *F*_2,4_ = 27.5, so the model allowing different levels of long-term priming in different sub blocks is inferior to one with a constant long-term priming effect throughout the neutral blocks. Thus, long-term priming was thus robust and did not decay when the feature bias was removed. The posterior distribution of $\mathcal {M}_{2}$ was in line with the assumption that short-term intertrial priming effects arose in this task (difference repetition- and switch effects: M =0.20, CI: [0.15,0.26]). Omitting repetition effects from $\mathcal {M}_{2}$ (yielding model $\mathcal {M}_{2-\text {rep}}$) resulted in *B* *F*_2,2−rep_>1000.

Repetition effects were found both before and after the bias, and are shown in Fig. 3D. Note that the size of the long-term priming effects is comparable to the size of the short-term priming effect. This is similarly indicated by the posterior of $\mathcal {M}_{2}$ (difference bias- and other effects: M = 0.16, CI: [0.11, 0.22]). We conducted a post-hoc exploration to investigate whether short- and long-term priming interacted. To this end, model $\mathcal {M}_{2}$ was extended with an interaction term for repetition and target color ($\mathcal {M}_{2+\text {rep}\times \text {tar}}$). The relative *B* *F*_2,2+rep×tar_ = 13.8, thus strongly supporting the absence of an interaction.

##### Accuracy

In Experiment 1B, overall accuracies were again very high (M = 97.2 %, SD = 2.3 %). The same analysis as for Experiment 1A yielded strong support for the absence of any accuracy effects, with $BF_{\textnormal {M}_{\text {null}}, 1} = 15.1$

##### Target color distribution estimates

Participants in Experiment 1B did, in general take note of the bias manipulation judging by their distribution estimates (M = 0.12, SD = 0.10; One-sided t-test: *B* *F*_+,0_>1000). Nevertheless, subjective experience of the trial distribution did not predict their resulting long-term priming effect. A Bayesian regression analysis, again, revealed no evidence for a correlation between distribution estimates and the long-term priming effect (*B* *F*_0,*β*_ = 4.4 considering all blocks, and *B* *F*_0,*β*_ = 4.8 considering only the last block). This suggested that long-term priming resulted from implicit memory, rather than an explicit strategy to prioritize search for either color.

### Discussion

In both Experiments 1A and B, we found strong effects of target repetition on search performance, with repetition of the target color resulting in shorter response times. However, in Experiment 1B, we additionally found strongly speeded responses to colors that had been biased. This long-term priming effect did not decay throughout a Neutral block of 200 trials, and did not appear to correlate with the extent to which participants subjectively experienced the bias. These two findings suggest the robust facilitation was not due to strategic prioritizing of either color. The findings from Experiment 1B correspond to the prediction from SAM (Fig. 1A), strongly suggesting that memory retrieval affected search as proposed by the episodic retrieval view. Surprisingly, however, in singleton search (Experiment 1A) there was no long-lasting memory influence; immediately after a bias block ended, the performance difference between both colors disappeared, in line with the (short-term) feature-weighting view (Fig. 1B).

What underlies this discrepancy? One possibility is that this finding extends the findings supporting hybrid accounts of priming, where it is proposed that episodic retrieval is a heuristic recruited when search is difficult (Lamy et al., 2011; Ásgeirsson and Kristjánsson, 2011). However, the different nature of the two search tasks may warrant a different explanation. In singleton search, targets are defined by deviating from the distractors, and can be found through ‘bottom-up’ feature guidance, regardless of the actual color of the target. In conjunction search, targets are never defined by merely deviating in a single dimension: the conjunction of features – in this case, being red or green, and diamond-shaped – is crucial to the task. Under these circumstances, ‘top-down’ attention is needed to identify the target. It might very well be that only when this exact identity of the target is relevant to the search process, memory traces that are laid down during the search process will differ for both target colors. If no differentiable memory traces are formed, as might be the case in singleton search, automatic retrieval of previous trial episodes would not facilitate the search for either color over the other, and thus not evoke a long-term priming effect.

Experiments 2A and B sought to disentangle which of these explanations could best account for the difference in the presence and absence of long-term priming effects observed here. Experiment 2A involved a variation of singleton search, which was rendered inefficient by using highly reduced color contrast and many more distractors than in Experiment 1A. Note that with such low color contrast targets will not ‘pop-out’ and search was expected to be inefficient. This would yield high RTs, but nevertheless the target’s identity was task-irrelevant. Experiment 2B was an easier variant of Experiment 1B, with less distractors more densely distributed over the display. In both experiments, the number of distractors was varied to produce a broad, overlapping range of RTs, as well as to explore the search efficiency in both tasks.

The results favored the view that the type of search, and not its difficulty, determined whether long-term priming was found: again, no long-term priming effects were found in singleton search, whereas they were present and persistent in the conjunction search task.

## Experiment 2A and 2B

### Method

#### Materials and stimuli

Displays for Experiment 2A, inefficient singleton search, were modeled after Experiment 1B (conjunction search), but differed in set size and stimulus colors. Set size was either 11 (three of each distractor shape, one distractor diamond and one target diamond) or 21 (six of each shape, two distractor diamonds and one target). In both set size conditions, the target was a singleton only in the color dimension. All distractors were of one color, and the target was a diamond with a deviant color. Two colors with low contrast (Fig. 2E) were determined by expressing ‘red’ and ‘green’ from Experiment 11 in HSI color space (0, 255, 161 and 120, 255, 101 respectively), equalizing intensity and reducing hue distance to 10 %, i.e. (54, 255, 101) for ‘red’ and (65, 255, 101) for ‘green’ (on a 0–255 scale).

In Experiment 2B, efficient conjunction search, displays were composed of the same stimuli as in Experiment 1B, with set sizes at either six or nine items. Specifically, each display contained the target diamond, one blue distractor diamond and four or seven non-diamond shapes, dependent on the set size. Every display thus contained two diamonds, all colors were balanced on each trial and each shape occured at least once (in the six-item condition) or twice (in the nine-item condition). Stimuli were randomly distributed over twenty-four equidistant locations on a circle at 11.23° eccentricity, with no stimulus immediately adjacent to another.

Participants again indicated whether the singleton colored diamond (in 2A) or the red or green diamond (2B) had a notch at the top or bottom. After the experiment, we again inquired about their experience of the bias manipulation.

#### Design and procedure

The design of both experiments was identical to that of Experiments 1A and B. As an additional consideration, the number of distractors (low or high) was counterbalanced across target colors within each block. Experiments 2A had five blocks, 2B had seven.

#### Participants

In Experiment 2A, data from 41 new participants were included (38 female, aged 18–27, M = 21.0, SD = 2.6, three participants discarded due to low accuracy), and 25 new participants were included in Experiment 2B (19 Female, aged 17–25, M = 20.9, SD = 2.5), excluding none. All participants were recruited from the same pool as those for Experiments 1A and B, and were given identical treatment.

#### Trial inclusion and color correction

Exclusion criteria for participants and trials were identical to those in Experiment 1A and B. This led to discarding 7.7 % of 600 trials in 2A (4.6 % errors and 2.5 % outliers) and 6.3 % in 2B (3.3 % errors and 2.4 % outliers). Analyses were again based on color-corrected standardized RTs.

#### Statistical analysis

The statistical models considered were the same as $\mathcal {M}_{1-4}$ used for Experiment 1A&B, but included an additional factor to explain the effect of the number of distractors (with levels low or high). For the accuracy-analyses we additionally included models $\mathcal {M}_{\text {null}}, \mathcal {M}_{\text {rep}}, \mathcal {M}_{\text {dist}}, \mathcal {M}_{\text {rep+dist}}$ to model the absence of effects, effects of repetition, the number of distractors or their combination, respectively. The same stopping criteria for data collection was used as in Experiments 1A and B.

### Results

#### Experiment 2A

##### Long- and short-term priming effects following a bias

Figure 4A illustrates the RTs for both target colors over the different blocks in Experiment 2A. The pattern of results is similar to that in Experiment 1A, in that the RT-benefits of the bias color seem restricted to the bias block, and do not carry over to the subsequent Neutral blocks. Similarly, the sub-block data shown in the inset does not support a persistent or slowly decaying facilitation within these Neutral blocks.

The analysis for Experiment 1A is in line with these observations: the two best fitting models were $\mathcal {M}_{1}$ and $\mathcal {M}_{2}$, with *B* *F*_2,1_ = 2.5 which suggests neither model to be highly preferred over the other. However, the posterior distribution for $\mathcal {M}_{2}$ suggests that zRTs were higher on bias-colored trials than on other-colored trials (difference bias- and other- effects, M =−0.04), contrary to the long-term priming hypothesis. One sided Bayesian t-tests on a facilitatory effect of the bias color ($\mathcal {M}_{\textnormal {bias}}$) versus a facilitatory effect of the other color ($\mathcal {M}_{\text {other}}$) versus no effect ($\mathcal {M}_{0}$), suggested a null-effect and argued against a long-term priming effect (*B* *F*_0,bias_ = 21.2,*B* *F*_0,other_ = 4.4). The posterior distribution of model $\mathcal {M}_{1}$ was furthermore in line with the assumed effects of short term priming, as repetition trials and low-distractor trials resulted in lower RTs (difference low and high number of distractor-effects: M =0.13, CI: [0.09,0.17]; difference repetition- and switch effects: M =0.30, CI: [0.26,0.33]). Omitting either effect from $\mathcal {M}_{1}$ yielded an $\mathcal {M}_{1-\textnormal {} x}$ with *B* *F*_1,1−*x*_>1000 for both factors. Repetition effects before and after the bias are illustrated in Fig. 4C.

##### Accuracy

Overall accuracies in experiment 2A were comparable to those in Experiment 1A&B (M = 95.7 %, SD = 3.4 %). The analysis on accuracies indicated that the best-fitting model was $\mathcal {M}_{\text {dist}}$, with *B* *F*_dist,rep+dist_ = 14.0, suggesting that only the number of distractors had an effect on accuracy. The posterior for $\mathcal {M}_{\text {dist}}$ was used to explore the effect size, and suggested participants performed slightly more accurate when the number of distractors was lower: (effect size: M =0.013, CI: [0.006,0.019]).

##### Target color distribution estimates

In Experiment 2A, there was no clear evidence whether participants overall took note of the bias manipulation (M = 0.03, SD = 0.18): a one-sided Bayesian t-test whether scores were greater than 0 yielded only limited evidence supporting overall awareness, with *B* *F*_0,+_ = 2.4. More importantly, and in line with Experiments 1A and B, distribution estimates did not predict the resulting long-term priming effect (*B* *F*_0,*β*_ = 5.7 considering all blocks, and *B* *F*_0,*β*_ = 5.6 considering only the last block).

#### Experiment 2B

##### Long- and short-term priming effects following a bias

For Experiment 2B, again the RTs for both target colors are plotted over blocks, in Fig. 4B. Despite a large overall decrease in reaction times, the pattern of results is largely similar to those of Experiment 1B, and the bias color shows persistent RT-benefits throughout the neutral blocks. Across the sub-blocks, again depicted in the inset, the average RTs-difference between both colors is largely consistent, although it might have slightly decreased in sub-block C.

In the analysis, the best fitting model is $\mathcal {M}_{2}$, highly preferred over all other models. This includes the second-best fitting model $\mathcal {M}_{4}$, with *B* *F*_2,4_ = 68.1 and, critically over model $\mathcal {M}_{1}$ with *B* *F*_2,1_>1000. In other words, the data offered strong support for the hypothesis of a long-term priming effect that again is persistent throughout the neutral blocks. The size of this long-term priming effect was indicated by the posterior (difference bias- and other- effects: M=0.09, CI: [0.06,0.13]). The hypothesized effects of short term repetition and distractor number were again supported as repetition trials and trials with fewer distractors produced lower RTs (difference low and high number of distractor-effects: M=0.37, CI: [0.33,0.41]; difference repetition- and switch effects: M=0.31, CI: [0.27,0.34]). Omitting either factor from model $\mathcal {M}_{2}$ yields a *B* *F*_2,0_>1000 for both these factors.

Short-term priming effects are depicted in Fig. 4D. Again, we explored whether long-term priming interacted with short term priming, or whether they were additive. Model $\mathcal {M}_{2}$ was extended with a term to express an interaction with repetition and target color ($\mathcal {M}_{2+\text {rep}\times \text {tar}}$). The *B* *F*_2,2+rep×tar_ = 6.9 offered fairly strong evidence that again, no interaction took place.

##### Accuracy

Overall accuracies in Experiment 2B were higher than in all other experiments, (M = 96.7 %, SD = 2.3 %). The analysis on accuracies supported an absence of any effects on accuracy, with $BF_{\textnormal {M}_{\text {null}}, \text {dist}} = 3.4$, and $BF_{\mathcal {M}_{\text {null}}, \text {rep}} = 21.5$, and higher BFs for comparisons with all other models.

##### Target color distribution estimates

Participants post-experiment distribution estimates in Experiment 2B were in general in line with the bias manipulation (M = 0.14, SD = 0.11), which was supported by the t-test (*B* *F*_+,0_>1000). The regression analysis again did not support the presence of a positive correlation between these scores and the resulting bias effects: (considering all blocks yielded minimal evidence for a *negative* slope. *B* *F*_*β*,0_ = 2.5,*M*_*β*_ = −0.88, CI: [−1.7,−0.2]; considering only the last block did not yield conclusive evidence: *B* *F*_0,*β*_ = 2.3).

#### Task difficulty and task types

Experiments 1A and B revealed a large discrepancy regarding the presence or absence of a long term priming effect. Experiments 2A and B sought to further investigate this discrepancy, and disentangle whether it could be attributed to the difference in task (singleton versus conjunction search) or due to differences in task difficulty or overall RTs. Experiments 2A and B involved a more difficult, inefficient search task and an easier conjunction search task. Both resulted in highly similar overall RTs, and yet long-term priming was only observed in conjunction search, not singleton search. Here, we present post-hoc analyses to further relate our findings to hybrid accounts of priming.

##### Episodic short-term priming effects

To explore whether our experiments yielded ‘episodic priming’ effects as they have been previously reported (Huang et al., 2004; Hillstrom, 2000; Lamy et al., 2011) we analyzed evidence for motor response repetition effects and interactions between motor response- and target repetition. For each experiment, we used the ‘best’ model found above as basis ($\mathcal {M}_{b}$), and extended it with terms for motor response repetition (m), an interaction between feature- and motor response repetition (f ×m) or both (m+f ×m), to yield models $\mathcal {M}_{\text {m}},\mathcal {M}_{\text {f}\times \text {m}}$, $\mathcal {M}_{\text {m}+\text {f}\times \text {m}}$. Again we only report comparisons between the best fitting models.

The results of Experiment 1A strongly supported the presence of an interaction (*B* *F*_f×m,*b*_>1000), yet not for a main effect of response repetition (*B* *F*_f×m,m+f×m_ = 5.9). In line with episodic priming effects, the interaction implied that feature repetition caused less facilitation when they were paired with response switch than a repetition, and vice versa (effect size M =0.10,CI: [0.07,0.14]). In Experiment 1B, no conclusive evidence on main effects of motor response repetition were found, (*B* *F*_*b*,m_ = 1.2), with in $\mathcal {M}_{m}$ somewhat faster responses to motor repetition trials (M =0.056,CI: [0.01,0.10]). The interaction was absent (*B* *F*_*b*,f×m_ = 11.5). In Experiment 2A, there was inconclusive evidence for a small main effect (*B* *F*_m,*b*_>2.1, effect size M =0.05,CI: [0.02,0.09]), but there was strong evidence that there was no interaction *B* *F*_m,m+f×m_ = 13.8 In Experiment 2B, there was very strong evidence for a main effect of response repetition M =0.10,CI: [0.07,0.14], M =0.10), but again not for an interaction (*B* *F*_m,m+f×m_ = 121.5). Surprisingly, these results are not in line with the predicted role of difficulty in these interactions: of these experiments, the singleton search task (1A) is clearly the easiest, and yet it is the only experiment that yielded interaction effects. In the other experiments, effects of motor response repetition were inconclusive or small, and independent from feature repetition.

##### Long-term priming effects and difficulty: response times

We further assessed to what extent difficulty affected the long-term priming effects found in this study. Of note, different definitions for ‘difficulty’ have been used in the literature: Lamy et al. (2011) noted the *overall* difficulty of the task expressed by RT determined whether participants utilized a strategy involving memory retrieval;, whereas Ásgeirsson and Kristjánsson (2011) suggested that *within* experiments, only trials with high RTs yielded episodic priming effects implying episodic retrieval.

To test whether long-term priming was sensitive to the overall level of difficulty of the search task we compared effect sizes from both conjunction search Experiments 1B and 2B, where long-term priming was found. We collapsed data from every neutral block following a bias for every participant, and computed the overall color-corrected zRT difference between the target colors after a bias to quantify the effect size.

Unfortunately, the data could not confirm nor reject whether the experiments differed in overall effect size (Bayesian one-sided t-test *B* *F*_0,+_ = 1.48).

**Fig. 5:**
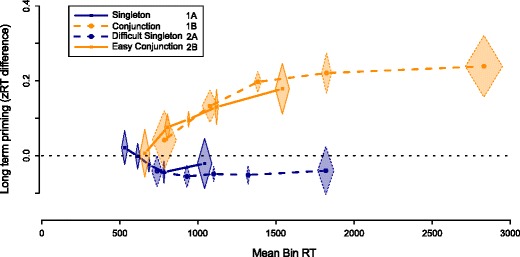
The effect of response time on the long-term priming effect. For each participant in each experiment, data was divided over five RT-bins. Data points reflect mean RTs in these bins and mean long-term priming effects in these bins. Shading indicates Cousineau-Morey 95% confidence intervals in both dimensions, computed separately per experiment. Long term priming effects are stronger with higher RTs, but only in the conjunction search tasks

Next, we explored whether RT differences within experiments affected long-term priming. Data from all neutral blocks following a bias block were collapsed and divided into five quantiles based on their RT. This was done separately for all four experiments. We then computed the zRT-difference to both target colors in each bin. The results are plotted in Fig. 5. This figure again illustrates the qualitative difference between conjunction search and singleton search: in both singleton search tasks, the difference between both target colors appears to remain constant across bins; in both conjunction search tasks, long-term priming is small when responses are quick, and gradually increases as RTs are slower. To quantify these trends, linear models were constructed, testing whether average RTs in each bin could predict effect sizes ($\mathcal {M}_{\beta }~\text {versus}~\mathcal {M}_{0}$). Indeed, both singleton search tasks showed evidence for the absence of a correlation (*B* *F*_0,*β*_ = 6.2 and 4.4 for Experiment 1A and 2A, respectively, *B* *F*_0,*β*_ = 9.3 when both experiments are taken together). Both conjunction search tasks, on the other hand, displayed evidence for such a correlation (*B* *F*_*β*,0_ = 4.0 for 1B and *B* *F*_*β*,0_ = 120*z*.5 for 2B, *B* *F*_*β*,0_ = 445.1 taken together). The slope coefficient *β*, estimated from the posteriors, differs between both experiments (CI: [1.9,11.7]×10^−5^ for 1B and [8.9,27.6]×10^−5^ for 2B), for which the straightforward reason might be that the slope of the curve in Fig. 5 appears to flatten for the highest RTs, which are much more common in Experiment 1B than 2B.

To summarize, the data from these four experiments do not directly support the view that difficulty determines strategic or heuristic involvement of memory affecting search. The quantile analysis again emphasized the clear difference between the conjunction- and singleton- search tasks: the latter showed no evidence for long-term priming effects, irrespective of the RT in each trial. The analysis did reveal that where long-term priming effects were found, they appeared to be larger with larger RTs. Nevertheless, further investigation would be necessary to determine whether this warrants an explanation based on difficulty: it could very well be that each overt and covert shift of attention in a trial was affected by long-term priming, and therefore trials that require more shifts show an amplified long-term priming effect.

##### Long-term priming effects and difficulty: efficiency

Another way to quantify search difficulty is through the search slope, or search efficiency. The posterior distributions for Experiments 2A and B suggest that adding more distractors had a much smaller effect on (z)RTs in the singleton search task (2A) than the conjunction search task (2B). We conducted two analyses to explore whether search efficiency would determine long-term priming. First, we conducted two bayesian regression analyses exploring the correlation between search slope and effect size for each participant ($\mathcal {M}_{\beta }~\text {or}~\mathcal {M}_{0}$) in Experiments 2A and B. These gave moderate evidence for the absence of a correlation in both (*B* *F*_0,*β*_ = 6.0 and 3.1, respectively).

Second, for Experiment 2A, we explored whether those participants who did engage in ‘true’ serial search – that is those who had a search slope of at least 10ms per item – would reveal any evidence of long term priming. We conducted the same model comparison as in our primary analyses on these participants. This did not lead to different conclusions: model $\mathcal {M}_{1}$ – with only short term repetition and the number of distractors as factors – was still strongly preferred over any model including an effect of target type (*B* *F*_1,2−4_>15.6). So although the sample size was relatively small (N = 9) the data still confirmed the absence of a long-term priming effect. Together, these analyses strongly suggest that when exploring search efficiency, again difficulty of the task does not affect long-term priming effects, as was similarly concluded for the analyses on overall RTs. Rather, the type of search task determines whether long-term priming effects are found.

## General discussion

Research has given rise to two very distinct views on how intertrial priming effects arise in visual search: short-term feature weighting or episodic retrieval of previous trials. Previous research on these accounts has mainly focused on whether features are primed independently or not. Here, we have investigated the different predictions both accounts make regarding the time course of priming. Using two computational models we derived differing predictions: only the episodic retrieval view predicts that when one target feature occurs more frequently for a number of trials, this can result in long lasting facilitation of this feature that persists throughout the experiment.

The four visual search experiments presented here directly tested these predictions. In each experiment, one of two possible target colors occurred on 80 % of trials during Bias blocks, while colors were equally likely in Neutral blocks. We found strong short-term intertrial priming effects in all experiments. Long-term priming was not evident in a typical singleton search task (Experiment 1A), but was clearly present in a conjunction search task (Experiment 1B): Bias color trials were faster than those of the other color. This effect did not decrease over the course of a 200-trial neutral block, suggesting a robust and persistent effect originating from long term memory. Our results argue against a view of this effect originating from an explicit strategy developed by participants, as we found evidence against a correlation between subjectively estimated bias and the resulting effect size. The effects were subsequently replicated with a more difficult singleton search task (Experiment 2A) and an easier conjunction search task (Experiment 2B). Although previous research has suggested that episodic retrieval of previous trials operates as a heuristic when search is ‘difficult’, this explanation alone could not account for our findings. Rather, the type of search (singleton or conjunction search) seems crucial to whether long term priming effects are found.

To our knowledge, this is the first time such a long lasting effect of a feature bias has been explored. Effects of spatial biases have been reported as statistical learning (Geng and Behrmann, 2005), but it has rarely been explored whether these effects persist after the bias is taken away: indeed, some have claimed statistical learning is at least partially, if not fully, the result of immediate, short term repetitions (Walthew and Gilchrist, 2006; Kabata and Matsumoto, 2012). Earlier explorations of the effects of feature biases on intertrial priming have focused on whether the predictability of repetitions and switch trials would modulate immediate repetition effects (Maljkovic and Nakayama, 1994, 2000; Maljkovic and Martini, 2005; Geyer and Müller, 2009). Many of these studies reported that increasing predictability had little to no effect on intertrial priming, supporting the short term independent feature weighting account. Geyer and Müller (2009) did find stronger immediate repetition priming when repetition of a feature was expected, and interpreted this as top-down modulation of feature weighting. The long term priming effect reported here, however, seems at odds with short-term independent feature weighting. However, it was readily predicted by episodic memory models: the feature bias establishes a bias in long term memory representations, which results in a persistent effect on search performance.

### Memory retrieval and Long-term effects in different types of search

A surprising aspect of our findings is that the long-term priming effect was limited to conjunction search, suggesting the effects of memory are qualitatively different for these two search types. Other evidence for this divergence is found in the literature: in the studies of Bichot et al. (1999) and Bichot and Schall (1999), macaques performed conjunction search for targets that were constant over a session. During those sessions, frontal eye field-neurons showed elevated responses to targets, as well as to distractors that shared features with the target. Eye movement patterns mirrored this neuronal selectivity. Crucially, similarly enhanced responses were also found to stimuli that shared features with the target from the previous session – which had been a day earlier. These results, mirroring our findings of long-term priming, were not found in identical experiments with a singleton search task; only short-term priming, up to 10 trials (Bichot et al., 1999).

What may underlie this divergence? As we have highlighted above, singleton search is generally thought to be driven by local comparisons between the target and the distractors, and relies on ‘bottom-up’ contrasts rather than the search for a particular target identity. From an episodic memory perspective, it could thus very well be that the memory traces that are formed in such bottom-up searches do not include a selection process based on a particular feature. For conjunction search tasks on the other hand, local feature comparisons are not sufficient – by definition some distractors will share features with the target. Rather, the target is defined by its identity (either a red or green diamond), and only an absolute match can lead to a successful search. In these tasks, search is driven by top-down guidance for either of the two targets. As a result, search with different target types may in this case lead to memory traces that include the target feature and affect future searches. The task thus shapes what is encoded in the memory traces. Such a view has also been advocated by Hommel (2004), Turk-Browne et al. (2005), and by Thomson and Milliken (2013).

This hypothesis would agree with studies that did report long term effects in bottom-up singleton search experiments. For example, Thomson and Milliken (2013) reported long-term contextual effects in singleton search, but only when a spatial configuration (the context) signaled that a different task was to be performed; when the spatial configuration was irrelevant to the task, no persistent effects were found (see also Thomson and Milliken, 2010). Another recent study (Becker et al., 2013) showed that if observers initially search for a specific color among *heterogeneous* distractors, this resulted in a persistent bias to attend stimuli with this color during a subsequent singleton search task. If, however, the first task was to search for this color among *homogeneous* distractors (i.e. singleton search), such long-term effects are not found. Thus, the target feature was encoded in memory traces when it was key to finding the target (in heterogeneous displays), but not when feature contrast alone was sufficient (in homogeneous displays). Note that the second task was always singleton search, suggesting that the task parameters at encoding – and not during retrieval – determined whether memory affected search. Leber et al. (2009) described a similar finding as the acquisition of an ‘attentional set’ to search for singleton features in general, versus searching for a particular unique feature.

Another effect that has been explained through associative retrieval of memory traces of previous trials, is the contextual cueing effect (Chun and Jiang, 1998, 2003). There, repetition of the spatial configurations of previously presented displays is found to facilitate search, through a bias for the target location. Typically, the contextual cueing effect is only reported with a conjunction search task. One study did find contextual cueing effects in singleton search (Geyer et al., 2010). Note, however, that in this study participants were pre-cued with the spatial configuration of the display 700ms before the stimuli appeared. Likely, this promoted the encoding of spatial information – producing contextual cueing – whereas in traditional singleton search, parallel local comparisons are sufficient.

These effects illustrate that visual search and attention are affected by long term memory, even when search tasks are driven by highly efficient bottom-up visual processing. Past history thus seems to persistently shape future deployments of attention, and thereby affects visual search. This perspective on search has interesting parallels with predictive coding theories of cognition and the brain (Rao and Ballard, 1999; Lee and Mumford, 2003; Friston, 2005). These theories propose the brain continuously seeks to predict what stimuli it will encounter, and that these predictions adjust perception and action – both at the behavioral and on the neuronal level. We argue that the formation and automatic retrieval of memory traces offers a mechanism to produce such predictions.

One particular finding supporting predictive coding theories is the Repetition Suppression (RS) effect found in fMRI-studies. RS entails that when a stimulus is repeated, the BOLD-response is found to be attenuated (Grill-Spector et al., 2006). A predictive coding view on this phenomenon states that our experience with a relatively static world leads the brain to expect stimuli to remain constant, i.e. to repeat. Subsequently, when this prediction of a repetition is met, this yields more effective processing of repeating stimuli relative to non-repeating ones (Summerfield et al., 2008; Summerfield and de Lange, 2014). To support this view, Summerfield et al. (2008) had participants attend to sequences of face stimuli, and manipulated the probability of repetitions in this sequence. They found that in blocks in which repetitions were frequent (and thus predicted more easily), the RS effect was larger than when stimuli mostly switched. A follow-up study by Larsson and Smith (2012) revealed that when the faces were not attended, clear RS was found both when repetitions were frequent as well as when they were infrequent. These findings have interesting parallels with the present study: First, it is the experience over the course of an entire block laid down in memory (the experienced ratios of repetitions and switches) that leads to these predictions. Second, how stimuli were processed during a trial –in their study whether they were attended or not; in the present study whether they required top-down guidance to be found – had a large effect on whether predictions were formed and whether traces laid down during search affected search in later trials.

### Automatic memory trace retrieval and intertrial effects

In short, episodic memory traces that affect visual search offer a way to account for when long term effects in visual search are found, and when they are not. But how does this relate to short-term intertrial priming? A role for episodic retrieval in intertrial priming was originally proposed to account for ‘episodic’ effects that a simple feature weighting account could not explain. Long-term priming, as reported here, would seem another one of such effects that cannot be explained by simple feature weighting, subject to swift passive decay (cf. Martini, 2010; Maljkovic and Nakayama, 2000). One could propose that there exists an additional long-term feature weighting mechanism. Such a proposal would require further elaboration on how such long-term feature weighting relates to short-term feature weighting, or why a discrepancy was found between singleton and conjunction search.

Nevertheless, although the findings from this study are readily explained by the episodic retrieval view, they do not invalidate a short term feature weighting mechanism. A parsimonious episodic retrieval account (as provided by the SAM model) explains both short- and long-term priming in one mechanism. However, in such a framework it is difficult to explain why in singleton search there are short-term but not long-term priming effects. Much like previous work that has favored a hybrid account of priming (Ásgeirsson and Kristjánsson, 2011; Lamy et al., 2011; Thomson and Milliken, 2013), we therefore feel both mechanisms may contribute to priming. Nevertheless, our results indicate that influences from episodic memory traces may be more prominent than previously thought, rather than that they are limited to late stages of difficult tasks.

Instead, we propose that the search task as a whole determines which priming mechanism dominates. In singleton search tasks, memory traces may not reflect a selection process based on particular features, and thus yield no priming effects on the long- or short term. There, priming effects largely result from short term feature weighting. In conjunction search tasks, however, the effects of feature weighting may be limited, as this will also render distractors that share features with the target more conspicuous. In these tasks retrieval of episodic memory traces may affect the search process and thereby evoke intertrial priming effects. Although the resulting intertrial effects in both tasks thus appear qualitatively similar, the underlying mechanism may very well be different.

## Conclusion

In this study, we have found short-term feature priming effects in both singleton- and conjunction search, but additional long-term priming effects only in conjunction search. Such durable long-term priming effects on top of short term priming are directly predicted by episodic memory models. We conclude that the the effects of episodic memory traces on visual search may be more consistent than previously thought, and that priming in conjunction search and singleton search may result in a large part from different mechanisms.

## Electronic supplementary material

Below is the link to the electronic supplementary material.

PDF 376 KB
