# Supplementary material for: The long and the short of priming in visual search
Source: Atten Percept Psychophys. 2015 Apr 2;77(5):1558–73. doi: 10.3758/s13414-015-0860-2 (PMC4468981; doi:10.3758/s13414-015-0860-2)
Supplement: Supplementary file 1 — PDF 376 KB [file 13414_2015_860_MOESM1_ESM.pdf]

The Long and the Short of Priming in Visual Search
Supplementary Material

Wouter Kruijne & Martijn Meeter
Vrije Universiteit Amsterdam

SAM as a model of visual priming

the SAM theory and model

"Search of Associative Memory", or SAM in short, is a theory of encoding and retrieval of items in memory. The SAM theory (presented in J. G. W. Raaijmakers & Shiffrin, 1981) aims to explain memory performance in a variety of experiments. We primarily based ourselves on the implementation of the theory that studied the dynamics of interference and forgetting (G.-J. Mensink & Raaijmakers, 1988; G.-J. M. Mensink & Raaijmakers, 1989). This model was designed to simulate findings from paired-associates list learning experiments, in which participants have to study word pairs (a – b). Later, in the test phase, participants are cued with one half of this pair, and have to recall the other (e.g. a – *?*). The model produces probabilities that an associate is recalled correctly, given one or several lists to be studied, maintenance intervals, and test lists of cues. The principles of SAM have successfully been applied to simulate free recall and recognition (for an overview J. G. Raaijmakers & Shiffrin, 2002).

SAM assumes that during encoding, stimulus information is associated and stored in long term memory as 'memory images', and partial information from an image can be used as a retrieval cue for the memorandum. For example the cue a – *?* is a retrieval cue for the memorandum $a - -b$. A critical assumption in SAM is that the memory image includes associations with a pool of contextual elements. In this pool, a limited subset of units is active at the same time, reflecting an 'episode' in memory. A subset of the elements active at the time of study is incorporated in the memory image, and contextual activity can thereby serve as a retrieval cue for a memorandum. The activity in the pool is subject to random drift: some of the active units become inactive over time and vice versa. These assumptions have two important implications that are also relevant for an episodic retrieval account of priming:

- (short-term) forgetting occurs gradually over time due to random drift of the context (as fewer and fewer of the units active at learning will be part of the context at retrieval.
- the more an item is studied, the more units will be associated with it. This increases the probability that it is retrieved, also at long retention intervals;

SAM as a model of visual priming by episodic retrieval

We used SAM as described in (G.-J. Mensink & Raaijmakers, 1988; J. G. W. Raaijmakers, 2003) and applied it to a typical visual search priming study. However, there are differences between the tasks that SAM was designed for ('memory tasks') and the way episodic retrieval may underly visual priming, which we took into consideration for our simulations. For example, memory tasks tend to have separate study and test phases which have participants actively study the items and 'search' for them in memory, respectively – two processes which can be assumed to be absent – or at least mostly implicit and passive – during a visual search task. Secondly, memory tasks involve a multitude of memoranda that can be easily individuated, and are recalled separately (e.g. in the paired associates task, the cue $a - -?$ has only one correct associate b). In the priming tasks simulated here, there are only two trial types (here: 'red' and 'green' trials). Without a well-defined individuating cue, so the retrieval of both trial types may simultaneously be probed throughout the trial [1]. Retrieval of memory traces laid down by any one of these two trial types will similarly have only one of two effects: the priming of either red or green targets. Finally, memory tasks challenge the participant to retrieve many different memoranda after a long delay. In priming in visual search, it is unlikely that participants will not be able to remember only two frequently presented target types. Therefore, it seems that for priming, the *relative* memory strength or ease of retrieval of the target types is crucial.

To simulate priming in visual search, we used the SAM model as outlined in (G.-J. Mensink & Raaijmakers, 1988; J. G. W. Raaijmakers, 2003) while accounting for these considerations:

- all parameters (Table 1) were given values adopted from previous SAM papers except one: since learning was no longer embedded in a study phase, we decreased the learning rate (w).
- we simulated the memory strengths of only two items, representing the two trial types ('red' or 'green') which were associated with the fluctuating context whenever they were presented. This allowed us to simulate priming for either trial type through the respective memory strengths.
- As a measure for priming effects caused by retrieval of memory traces, we computed the probability of retrieval for both memory images. Then we defined the amount of facilitation by a memory item (F^i), as its recall probability divided by the sum of both recall probabilities. Since there are no explicit retrieval cues, this probability of recall is determined by the contextual cues, and priming is thereby determined by the bias in memory. Note that this measure is very similar to 'global familiarity' as was introduced to simulate recognition tasks with SAM (Gillund & Shiffrin, 1984).

[1] One could argue that the onset of a trial constitutes a retrieval cue, or that other aspects of a trial probe retrieval, for example the stimulus layout. The latter certainly seems to be the case with the contextual cueing effect (Chun & Jiang, 1998, ,discussed in the main article discussion). Similarly, the episodic retrieval account explains 'episodic priming' effects through better retrieval when all visual features match the previous trias compared to when they do not (Hillstrom, 2000; Huang, Holcombe, & Pashler, 2004). For the present simulations, however, we focus on priming of pop-out tasks (Maljkovic & Nakayama, 1994) where trials only differ in their color, and all other aspects of the tasks are balanced.

Table 1
Parameters used in SAM simulations of priming experiments

Parameter	Value	Description
α	0.08	Drift rate parameter
s	0.2	Ratio of active/inactive units in the LTS
a	5.0	Scaling constant for context association strength
w	0.75	learning rate, is 1.0 in most SAM simulations
Z	3.0	Interference from other memory traces at retrieval
t_p	1.5	Duration of stimulus presentation/encoding (seconds)

- We had no explicit hypothesis how memory retrieval influences RTs throughout the search trial. We assumed that this influence could be task- and participant-specific, and that the influence would saturate at a certain level. Therefore we fitted the following function to experiment data:

$$RT = c + ge^{-\tau F^i}$$

Where c reflects a baseline RT when priming is maximally effective, assuming that memory influence can not facilitate RTs below a asymptotic minimum; g scales the priming effect, and the exponential term describes how fast priming saturates (τ). Note that Figure 1 in the article depicts F^i rather than simulated RTs.

We simulated data from three experiments:

- Maljkovic and Nakayama (1994, Figure 7, bottom): Maljkovic and Nakayama reported the average RTs of each trial on which the target had the same versus a different color N trials in the past. The data is from one naive participant, and illustrates how priming gradually decays over the course of multiple trials. The simulations reveal the same pattern, although priming decays somewhat faster.

- Brascamp, Pels, and Kristjánsson (2011, Figure 1D): the time course of priming was probed by exploring how facilitation 'accumulates' over multiple repetitions of same-colored build-up trials, then 'breaks down' over intervening trials of the other color. measured by the RT on one final trial of the build-up color. Data comes from from six participants. Especially with few intervening trials, different build-up conditions show different priming.

- Martini (2010, Figure 5, left): Martini formalized short-term decay in priming, combining z-scored RTs from 50 participants, and computing the average contribution of trials matching in color N trials back. The facilitation (through priming) of each trial in the past again decays over several trials. The SAM simulations very closely match the empirical data, illustrating that both models are comparable under these conditions where target types are balanced.

All simulated experiments were repeated 25 times to reflect multiple participants, and all runs were preceded by a 'training phase' where 20 trials – 10 of each type – were

presented in random order. The simulations of the data from Maljkovic and Nakayama; Martini were then followed by 500 trials, balanced for both colors, in random order. For the data of Brascamp et al., each combination of buildup and intervening trials was ran, separately. The plots of the experiment data and the SAM-simulation data are given in figure 1. All three experiments were simulated very well by the model.

Raw Response Times

The plots in the paper all reflect color corrected response times. Here, we visualize the average raw RTs, in Figure 2.

References

Brascamp, J. W., Pels, E., & Kristjánsson, Á. (2011). Priming of pop-out on multiple time scales during visual search. *Vision research*, *51*(17), 1972–1978.

Chun, M. M., & Jiang, Y. (1998). Contextual cueing: Implicit learning and memory of visual context guides spatial attention. *Cognitive psychology*, *36*(1), 28–71.

Gillund, G., & Shiffrin, R. M. (1984). A retrieval model for both recognition and recall. *Psychological Review*, *91*(1), 1–67.

Hillstrom, A. P. (2000). Repetition effects in visual search. *Perception & Psychophysics*, *62*(4), 800–817.

Huang, L., Holcombe, A. O., & Pashler, H. (2004). Repetition priming in visual search: Episodic retrieval, not feature priming. *Memory & Cognition*, *32*(1), 12–20.

Maljkovic, V., & Nakayama, K. (1994). Priming of pop-out: I. role of features. *Memory & cognition*, *22*(6), 657–672.

Martini, P. (2010). System identification in priming of pop-out. *Vision research*, *50*(21), 2110–2115.

Mensink, G.-J., & Raaijmakers, J. G. W. (1988). A model for interference and forgetting. *Psychological Review*, *95*(4), 434.

Mensink, G.-J. M., & Raaijmakers, J. G. W. (1989, June). A model for contextual fluctuation. *Journal of Mathematical Psychology*, *33*(2), 172–186.

Raaijmakers, J. G., & Shiffrin, R. M. (2002). Models of memory. In J. Wixted & H. Pashler (Eds.), *Stevens' handbook of experimental psychology.* Wiley.

Raaijmakers, J. G. W. (2003). Spacing and repetition effects in human memory: Application of the sam model. *Cognitive Science*, *27*(3), 431–452.

Raaijmakers, J. G. W., & Shiffrin, R. M. (1981). Search of associative memory. *Psychological review*, *88*(2), 93.

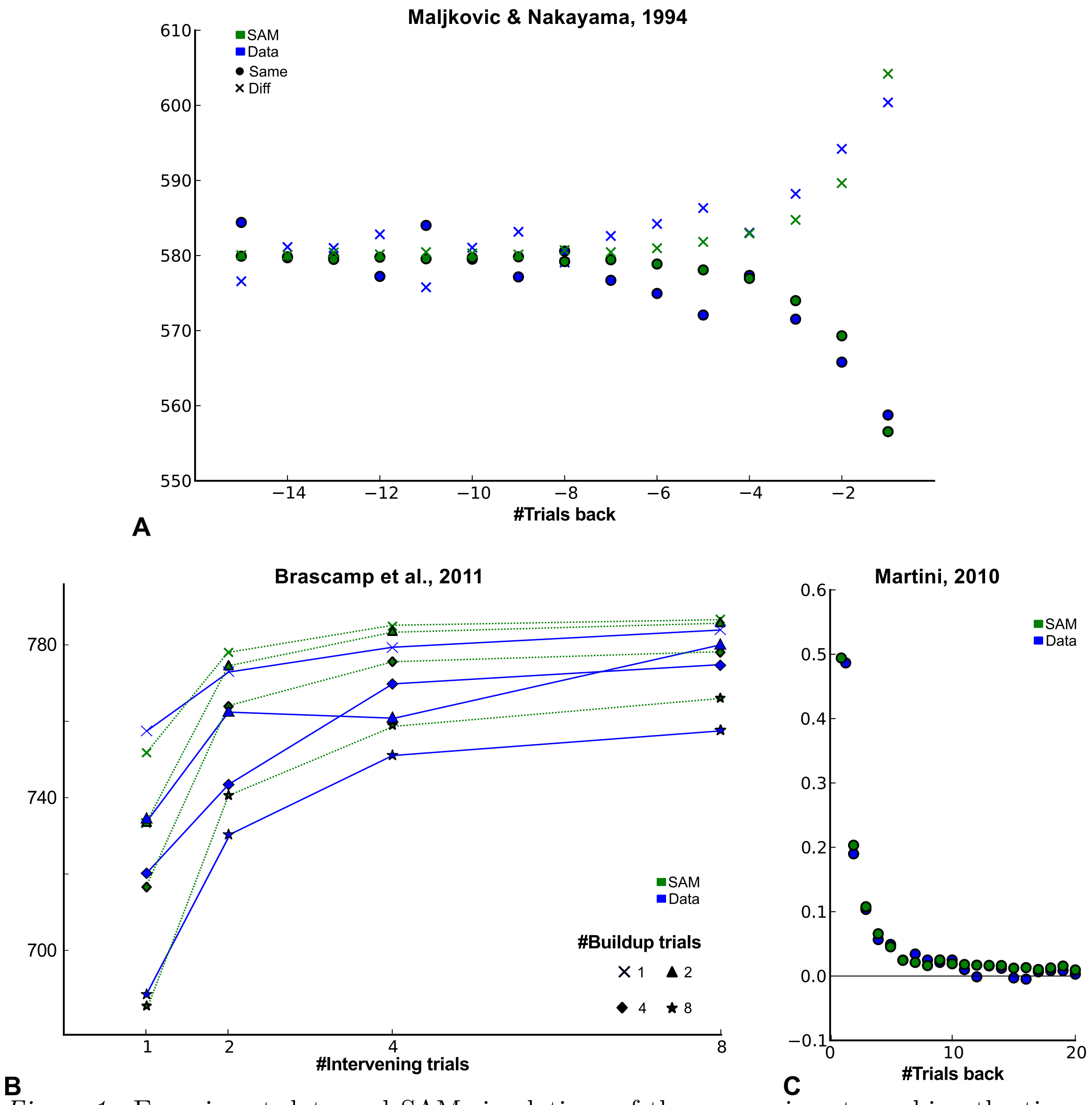

Figure 1. Experiment data and SAM simulations of three experiments probing the time-course of intertrial priming (see text for details) **A** the average RT of trials that have the same target color as the target on 1 – 15 trials back (Maljkovic & Nakayama, 1994). **B** the average RT of trials of one particular color after a specified number of 'buildup' trials of the same color and a number of 'intervening' trials of the opposing color (Brascamp et al., 2011). **C** the facilitative effect evoked by a trial repetition from n trials in the past, computed from z-scored response times from 50 participants (Martini, 2010).

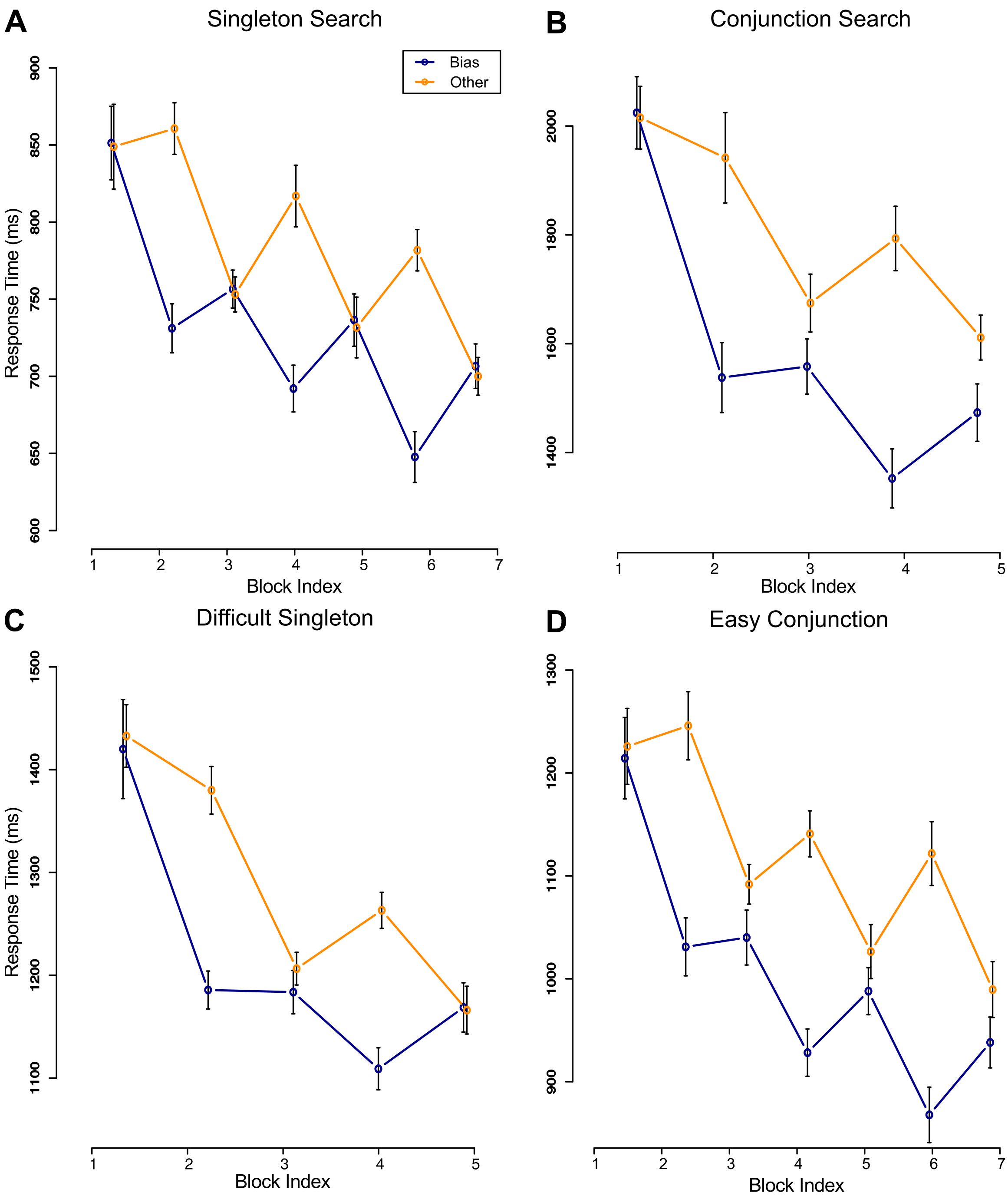

Figure 2. Data from the four experiments without correcting for observers' *a priori* color differences. The odd-numbered block indices reflect the neutral blocks. Bars reflect 95% Cousineau-Morey confidence intervals **A** Experiment 1A. **B** Experiment 1B. **C** Experiment 2A. **D** Experiment 1B.
